# Complementary Experimental Methods to Obtain Thermodynamic Parameters of Protein Ligand Systems

**DOI:** 10.3390/ijms232214198

**Published:** 2022-11-17

**Authors:** Shilpa Mohanakumar, Namkyu Lee, Simone Wiegand

**Affiliations:** 1 IBI-4—Biomacromolecular Systems and Processes, Forschungszentrum Jülich GmbH, D-52428 Jülich, Germany; 2 Chemistry Department-Physical Chemistry, University of Colgone, D-50939 Cologne, Germany

**Keywords:** thermophoresis, thermodiffusion, Soret effect, protein-ligand binding, hydration effects, entropy–enthalpy compensation, thermal diffusion forced Rayleigh scattering, isothermal titration calorimetry, thermophoretic microfluidic cell

## Abstract

In recent years, thermophoresis has emerged as a promising tool for quantifying biomolecular interactions. The underlying microscopic physical effect is still not understood, but often attributed to changes in the hydration layer once the binding occurs. To gain deeper insight, we investigate whether non-equilibrium coefficients can be related to equilibrium properties. Therefore, we compare thermophoretic data measured by thermal diffusion forced Rayleigh scattering (TDFRS) (which is a non-equilibrium process) with thermodynamic data obtained by isothermal titration calorimetry (ITC) (which is an equilibrium process). As a reference system, we studied the chelation reaction between ethylenediaminetetraacetic acid (EDTA) and calcium chloride (CaCl${}_{2}$) to relate the thermophoretic behavior quantified by the Soret coefficient $S_{T}$ to the Gibb’s free energy ΔG determined in the ITC experiment using an expression proposed by Eastman. Finally, we have studied the binding of the protein Bovine Carbonic Anhydrase I (BCA I) to two different benzenesulfonamide derivatives: 4-fluorobenzenesulfonamide (4FBS) and pentafluorobenzenesulfonamide (PFBS). For all three systems, we find that the Gibb’s free energies calculated from $S_{T}$ agree with ΔG from the ITC experiment. In addition, we also investigate the influence of fluorescent labeling, which allows measurements in a thermophoretic microfluidic cell. Re-examination of the fluorescently labeled system using ITC showed a strong influence of the dye on the binding behavior.

## 1. Introduction

Quantification of biomolecular interactions is extremely valuable in applications such as drug discovery and understanding molecular disease mechanisms. Several techniques, such as Bioluminescence Resonance Energy Transfer (BRET) [1], Atomic Force Microscopy (AFM) [2], and Fluorescence Cross-Correlation Spectroscopy (FCCS) [3], have been developed providing binding affinities, kinetics, and/or thermodynamics of the interactions [4]. One of the newer methods is MicroScale thermophoresis (MST) [5]. MST measures the thermophoretic movement of solutes in a temperature gradient by recording the fluorescent intensity. Typically, the binding constant is derived by using multiple capillaries with constant concentrations of protein and increasing ligand concentration. The capillaries are scanned consecutively, so that $K_{a}$ can be determined, which gives access to the change in Gibb’s free energy ΔG and has been demonstrated in a series of experiments [6,7]. Since the technique uses fluorescent detection, either a fluorescent label is attached or the inherent fluorescence of the molecule of interest is detected [8,9]. The fluorescent labeling is very selective and allows low concentrations, but on the other hand the fluorescent label might influence the binding of the ligand. Although the underlying measurement effect is thermophoresis, the Soret and thermal diffusion coefficients are not determined in the commercial instrument, but this will be possible with a modified set-up [10].

Thermodiffusion is quantified by the Soret coefficient $S_{T}=D_{T}/D$, with the thermal diffusion coefficient $D_{T}$ and the diffusion coefficient *D* [11,12]. A negative $S_{T}$ indicates thermophilic behavior which means the solute accumulates on the warmer side. While $S_{T}$ being positive (thermophobic) indicates a movement of the solute towards the colder side. Studies of aqueous systems suggest that the change in the thermodiffusive behavior is often connected with a variation in the hydration shells [13,14,15]. For certain solutions, a sign change from thermophilic to thermophobic behavior can be observed at a transition temperature $T^{*}$ [16]. An empirical equation for diluted aqueous solutions proposed by Iacopini and Piazza [17] describes the temperature dependence by,

(1) $$S_{T}\left(T\right)=S_{T}^{\infty }\;\left[1-\exp\left(\frac{T^{*}-T}{T_{0}}\right)\right]\;,$$

where $S_{T}^{\infty }$ is a constant value approached at high temperatures, $T^{*}$ is the temperature at which the sign change of $S_{T}$ occurs and $T_{0}$ indicates the curvature. Equation (1) describes how $S_{T}$ increases with increasing temperature: $S_{T}$ is low at lower temperatures approaching a plateau value at high temperatures [13,14,15]. Solute-solvent interactions play a crucial role in the temperature sensitivity of $S_{T}$. In aqueous solutions, this contribution decreases with rising temperature due to breaking of hydrogen bonds [18]. For a number of aqueous systems, the difference of $S_{T}$ at two different temperatures $\Delta S_{T}$ has found to correlate with log *P* (partition coefficient) [14,19]. This indicates that the hydrophilicity of the solute plays a crucial role in the thermophoretic behavior of aqueous systems. Log *P* or the partition coefficient describes the concentration distribution of a solute between an aqueous and a 1-octanol phase in equilibrium. Thus, *P* is defined as

(2) $$P=\frac{{\left[\mathrm{solute}\right]}_{\mathrm{octanol}}}{{\left[\mathrm{solute}\right]}_{\mathrm{water}}}$$

Solutes which are highly hydrophilic (low or negative log *P*) show a stronger change of $S_{T}$ with temperature compared to more hydrophobic solutes [14]. At low temperatures hydrophilic solutes form many hydrogen bonds with water, while their number and strength decrease with increasing temperature. This means that at lower temperature there is a greater change in the hydration layer, which affects the Soret coefficient to a greater extent [20,21,22].

To investigate the thermophoretic behavior quantitatively we use Thermal Diffusion Forced Rayleigh Scattering (TDFRS). This is an optical method which analyzes the diffraction efficiency of a refractive index grating due to temperature and concentration modulation. Ideally, the method is applied to binary mixtures, so biological systems with several components (buffer compounds) to stabilize the solution are more challenging because all compounds contribute to the refractive index contrast and complicate the analysis. So far only the strongly binding protein–ligand system streptavidin with biotin has been studied by TDFRS [18,23]. The measurements were supported by neutron scattering experiments and also isothermal titration calorimetry data were included in the analysis [24,25]. Experiments showed that the temperature sensitivity of the Soret coefficient was reduced for the complex compared to the free protein indicating that the complex was less hydrophilic leading to a larger entropy of the hydration layer. The outcome agreed with neutron scattering data. The study of this particular system illustrates that thermodiffusion and its temperature dependence are highly sensitive to changes in the hydration layer. Although the exact mechanism of these changes cannot be evaluated by the study of a single system, measurements of similar systems can give us a more explicit picture on the conformational and hydration changes that occur upon ligand binding.

Isothermal titration calorimetry (ITC) is a standard method for any chemical (binding) reaction [26]. It directly measures the heat released or consumed in the course of a molecular binding event. In addition to thermodynamic parameters, such as enthalpy ΔH, entropy ΔS, and Gibb’s free enthalpies ΔG change, the equilibrium-binding affinity $K_{a}$ and interaction stoichiometry can be determined. Among the biophysical characterization methods ITC offers the highest information content [4].

A recently developed thermophoretic microfluidic cell was so far only tested with fluorescently labeled colloidal particles [27]. In principle, the cell can also be used to monitor quantitatively the thermophoretic properties of fluorescently labeled free proteins and complexes as used in MST.

Although the thermophoretic behavior of the free protein compared to the protein–ligand complex differs, the microscopic mechanism for this change is not yet understood. The underlying physical effect is one of the interesting unsolved puzzles in physical chemistry. Binding reactions are quite complex, strongly influenced by several factors, such as temperature, concentration, pH, ionic strength, etc., and, in turn, influence the thermophoretic motion [11,12,28]. In this work, due to the complexity of the system and the physical effect, we study chemical binding reactions with TDFRS and ITC. Based on the results of the complementary methods we want to establish a relation between thermodynamic parameters obtained by ITC and thermophoretic properties measured with TDFRS. Additionally, selective ITC measurements and studies in a thermophoretic microfluidic cell were performed to investigate the influence of a fluorescence label on the binding and thermophoretic behavior.

To connect the thermodynamic parameter determined with ITC with the non-equilibrium coefficient derived from TDFRS experiments, we start from an early work by Eastman [29]. In modern notation, his approach connects the Soret coefficient, $S_{T}$, to the Gibb’s free energy as follows [29,30]:

(3) $$S_{T}=\frac{1}{k_{B}T}\frac{dG}{dT}$$

This approach is not viewed uncritically, already de Groot wrote [31], that Eastman’s theory is “…certainly not rigorous at all”. Integrating Equation (3) with respect to temperature will give us access to a relation between $S_{T}$ and ΔG for the individual compounds of the system (free protein, free ligand, and complex). A detailed derivation can be found in the Supporting Information Section S1.

**Figure 1:**
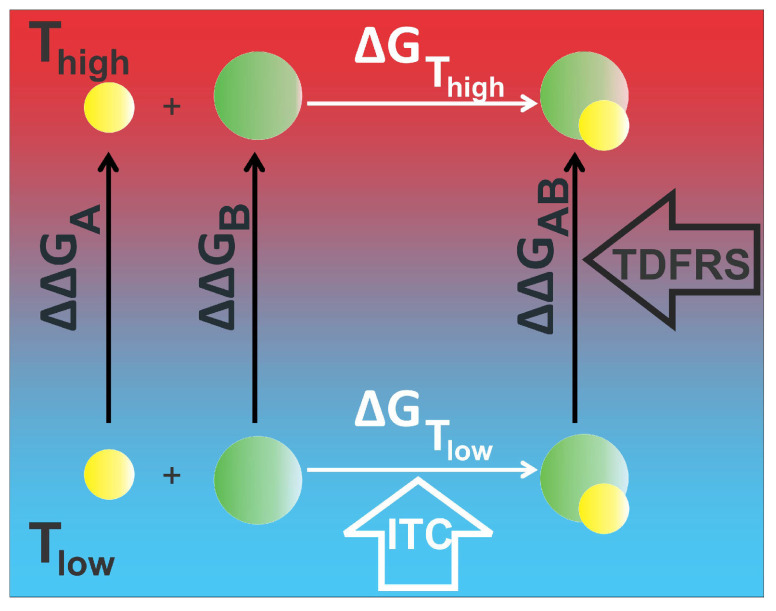
Schematic illustration of the calculation of ΔG and ΔΔG from ITC and TDFRS, respectively.

How these individual contributions can be used to establish a relation between ITC and TDFRS measurements is illustrated in Figure 1. “A” and “B” correspond to the molecules which are used to form the complex “AB”. We measure the free energy change ΔG at two different temperatures with ITC ($\Delta G_{T_{\mathrm{low}}}$ and $\Delta G_{T_{\mathrm{high}}}$). We hypothesize that $\Delta G_{T_{\mathrm{high}}}$ can be calculated from the free energy change at low temperature $\Delta G_{T_{\mathrm{low}}}$ measured by ITC and the differences in ΔΔG corresponding to two temperatures for the individual components probed by TDFRS using the following equation:

(4) $$\Delta G_{T_{\mathrm{high}}}=\Delta G_{T_{\mathrm{low}}}+\Delta \Delta G_{\mathrm{AB}}-\Delta \Delta G_{A}-\Delta \Delta G_{B}.$$

**Figure 2:**
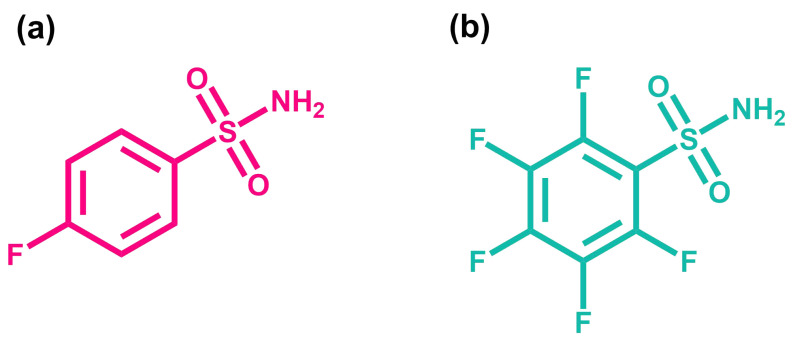
Chemical structure of the two investigated ligands: (**a**) 4-fluorobenzenesulfonamide (4FBS) and (**b**) Pentafluorobenzenesulfonamide (PFBS).

To test our hypothesis, we use EDTA and CaCl${}_{2}$ in MES buffer as reference system. The chelation reaction between ethylenediaminetetraacetic acid (EDTA) and calcium chloride (CaCl${}_{2}$) is a well known reaction which is used as a validation standard for ITC measurements [32]. EDTA exists in several forms in MES buffer [33,34,35]. Details of the existing forms (Figure S1a,b) can be found in the Supporting Information Section S2. In the next step, we use the same formalism for the protein Bovine Carbonic Anhydrase I (BCA I) with two different ligands. The enzyme BCA I is responsible for the conversion of carbon dioxide to bicarbonate [36] and inhibitors of this enzyme are used for the treatment of glaucoma and epilepsy [37]. Arylsulfonamides have the highest affinity and are mainly used as inhibitors for BCA I [38,39]. In our study, we used 4-fluorobenzenesulfonamide (4FBS) and Pentafluorobenzenesulfonamide (PFBS) (cf. Figure 2). A previous study of BCA II, which is a variant of our enzyme, shows that PFBS binds approximately 25 times stronger than 4FBS at 25 °C [40,41]. Therefore, we assume that the binding for these two ligands differs for BCA I as well, so that we can test our method for varying binding constants.

## 2. Results and Discussion

### 2.1. EDTA–CaCl${}_{2}$ System

#### 2.1.1. TDFRS Measurements

**Figure 3:**
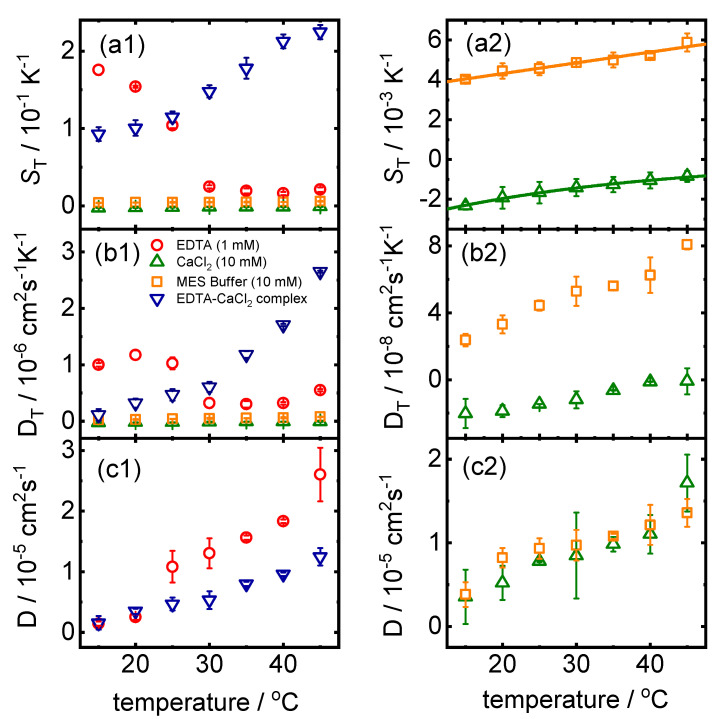
Temperature dependence of (**a1**) $S_{T}$ and (**b1**) $D_{T}$ for EDTA (1 mM), CaCl${}_{2}$ (10 mM), MES buffer (10 mM), and EDTA–CaCl${}_{2}$ complex. Figures on the corresponding right panel is a zoomed in image of temperature dependence of (**a2**) $S_{T}$ and (**b2**) $D_{T}$ for CaCl${}_{2}$ and MES buffer. (**c1**,**c2**) show the temperature dependence of *D* for EDTA, EDTA–CaCl${}_{2}$ complex and CaCl${}_{2}$, MES buffer, respectively. The error bars correspond to the standard deviation of the mean of repeated measurements. Lines in (**a2**) corresponds to a fit according to Equation (1).

We conducted IR-TDFRS measurements for the individual components EDTA, CaCl${}_{2}$, MES Buffer, and EDTA–CaCl${}_{2}$ complex. Figure 3 shows the temperature dependence of $S_{T}$, $D_{T}$, and *D* for EDTA (1 mM), CaCl${}_{2}$ (10 mM), MES buffer (10 mM), and EDTA–CaCl${}_{2}$ complex. Temperature dependence of ${\left(\partial n/\partial c\right)}_{p,T}$ and ${\left(\partial n/\partial T\right)}_{p,c}$ used to analyze thermophoretic measurement for each temperature is shown in Supporting Information (Figure S5).

$S_{T}$ of MES buffer is positive, while CaCl${}_{2}$ in buffer displays thermophilic behavior ($S_{T}<0$). For both systems, the temperature dependence of $S_{T}$ can be described by Equation (1). The Soret coefficient of MES buffer and CaCl${}_{2}$ in buffer is of the order of $10^{-3}$ K${}^{-1}$, while $S_{T}$ of EDTA and the complex EDTA–CaCl${}_{2}$ are two orders of magnitude larger (cf. Figure 3(a1)). Therefore, we treat the solutions of EDTA and the complex (EDTA–CaCl${}_{2}$) as a quasi-binary system analyzing the TDFRS data. The Soret coefficient of the complex shows an increase with temperature, but cannot be described by Equation (1) as it has a turning point. $S_{T}$ of EDTA decays with increasing temperature with an unusual pronounced drop between 25 °C and 30 °C. In the literature [42,43,44,45], there are works reporting a change of behavior in properties of several systems in presence of EDTA around 30 °C compared to that of room temperature, but so far no explanation has been developed. A similar sudden change of $S_{T}$ with temperature in the same temperature range has been reported for poly(*N*-isoproplacrylamide) (PNiPAM) in water [46]. PNiPAM is a temperature sensitive polymer showing a coil globule transition between 25 °C and 33 °C [47,48]. A small part of the drop of $S_{T}$ is related to the increase in the diffusion coefficient, but the larger part is caused by the abrupt drop of $D_{T}$ when the polymer coil collapses [46]. In the case of EDTA as well, the diffusion coefficient shows an abrupt increase between 20 °C and 25 °C, which is 5 K lower than the abrupt drop of $S_{T}$ and $D_{T}$. The mechanism leading to the change in $S_{T}$, $D_{T}$, and *D* of EDTA in water might have the same origin as in the case of PNiPAM as it happens in a similar temperature range so that it is very likely influenced by hydrogen bonds. Bischofsberger et al. [48] argue that at higher temperatures the system minimizes its free energy by gaining entropy through the release of water molecules from the hydration shell. Although the microscopic mechanism is still unclear, this is further evidence that changes in water structure affect thermophoretic motion.

We notice that the diffusion data of EDTA and EDTA–CaCl${}_{2}$ agree at low temperatures, while they differ clearly at higher temperatures. This might indicate a similar hydrophilicity of EDTA and the complex at low temperatures. Further, we observe, that the diffusion coefficients of MES buffer and the CaCl${}_{2}$ (10 mM) agree in both cases, as these are small molecules with similar diffusion behavior.

#### 2.1.2. ITC Measurements

As mentioned before, EDTA–CaCl${}_{2}$ is a system that has been well studied and characterized using ITC at room temperature [32,49,50]. For our goal we need binding parameters of the system in a wide temperature range. Our results are summarized in Table 1 and an example of a typical ITC measurement of the EDTA–CaCl${}_{2}$ binding reaction at 25 °C is shown in Figure S7.

The reaction is found to be temperature sensitive and is more favored at lower temperatures. This is similar to what has been observed by Arena et al., monitoring the association constant of the exothermic reaction between EDTA and Ca${}^{2+}$ [51], where they found a decrease with increasing temperature.

### 2.2. Protein–Ligand System

#### 2.2.1. TDFRS Measurements

**Figure 4:**
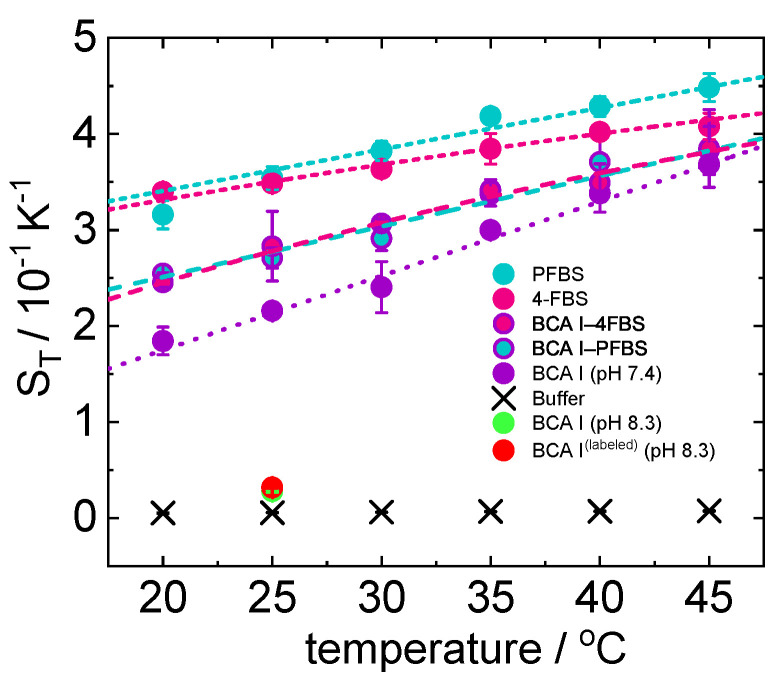
Temperature dependence of $S_{T}$ for BCA I (10 μM, pH 7.4, violet bullets), BCA I (10 μM, pH 8.3, green bullets), labeled BCA I (pH 8.3, red bullets), 4FBS (110 μM, pink bullets), and PFBS (110 μM, turquoise bullets), corresponding protein–ligand complex, sodium phosphate buffer (20 mM, Black cross).

Temperature dependence of the thermophoretic behavior of the free protein (BCA I), free ligands (4FBS and PFBS), and protein–ligand complexes is shown in Figure 4. Change of *D* and $D_{T}$ with temperature for the free protein (BCA I), both ligands (4FBS and PFBS), and protein–ligand complexes is shown in the Supporting Information (cf. Figures S3 and S4). Temperature dependence of ${\left(\partial n/\partial c\right)}_{p,T}$ and ${\left(\partial n/\partial T\right)}_{p,c}$ used to analyze thermophoretic measurement for each temperature is shown in Supporting Information (cf. Figure S6). As expected, the Soret coefficient $S_{T}$ of free BCA I changes significantly once the ligand binds. $S_{T}$-values of the complexes BCA I-4FBS and BCA I–PFBS are higher compared to that of the free protein. It can also be noticed that $S_{T}$ of both complexes are indistinguishable within the error bars. This means that the hydration shells of the complexes formed are very similar, but different from those of the free protein. Increase in $S_{T}$ with temperature of BCA I-ligand complex compared to free BCA I is different from that observed for the Streptavidin-biotin (STV-B) system [23]. For STV-B the difference between $S_{T}$ of the free protein and complex increases with increasing temperature. This was attributed to the stiffness of the protein at low temperatures so that the binding of the ligand (biotin) has a weaker effect at these temperatures [23]. In contrast to this, for both protein–ligand systems that we have studied the difference between $S_{T}$ of free protein and complex decreases with temperature, so that it is almost negligible at high temperatures. This is an indication that the binding of both the ligands should become weaker with increasing temperature. This is in line with ITC measurements, which will be discussed in detail in Section 2.2.2.

Hydrogen bonds have a clear influence on the variation of $S_{T}$ with temperature. Change in $S_{T}$ with temperature is more evident, if the solute can form more hydrogen bonds with water [12,23], therefore, we conclude that the free protein is more hydrophilic than the protein–ligand complex (cf. Figure 4). So far, the temperature dependence of the thermophoretic behavior has only been studied for two other binding systems; STV–B and various unmethylated cyclodextrins with acetylsalicylic acid [19,23]. In both cases, the stronger temperature dependence of the free protein or host molecule indicates a lower hydrophilicity of the formed complexes.

As we could not find studies which looked into the reaction mechanism of BCA I with the selected sulfonamide ligands, we compared Human Carbonic Anhydrase I (HCA I) with 4FBS and Bovine Carbonic Anhydrase II (BCA II) with both ligands which have been well characterized [40,52,53,54,55]. The active site of the different variants of carbonic anhydrase protein (HCA I, BCA II) is the Zn${}^{2+}$ ion that is tetrahedrally coordinated by three histidyl residues and a water molecule [56,57], to which sulfonamide ligands usually bind [39,58]. In the literature, two scenarios of binding of sulfonamide ligands are discussed. The first suggests that sulfonamides are present in the anionic form in their complexes with carbonic anhydrase [40,53,54,55,59], while the latter proposes neutral sulfonamides are bound to the active zinc ion [53]. The detailed mechanism in both the cases has been discussed by Krishnamurthy et al., [40]. It has to be noted that in both possible scenarios a water molecule is being released upon ligand binding. This implies that the complex is less hydrophilic than that of the free protein, which is what has also been concluded from the thermophoretic data.

In the literature, it has been reported that an increase in fluorination decreases the strength of hydrogen bond network between SO${}_{2}$NH group and the active site of the target protein [40]. This implies that the complex of BCA I with PFBS (which is highly fluorinated) should show a weaker temperature sensitivity of $S_{T}$ compared to 4FBS. This is what we observe from our TDFRS measurements as we find; $\Delta S_{T}$ = $S_{T}$(45 °C) − $S_{T}$(20 °C), $\Delta S_{T}$(BCAI–4FBS) = 0.139 K${}^{-1}$ and $\Delta S_{T}$(BCAI–PFBS) = 0.128 K${}^{-1}$.

#### 2.2.2. Isothermal Titration Calorimetry Measurements

**Figure 5:**
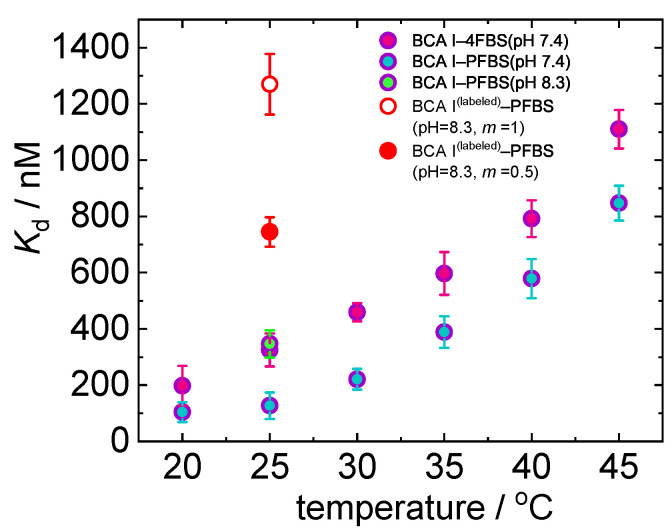
Temperature dependence of K${}_{d}$ for BCA I-4FBS and BCA I–PFBS complexes measured with ITC at pH = 7.4. For comparison we show also a single measurement at 25 °C of the labeled and unlabeled BCA I–PFBS complex at pH = 8.3. For the labeled BCA I–PFBS complex, we report two K${}_{d}$ values; red open circle (value that is obtained with m=1) and red closed circle (value that is obtained with m=0.5). More details about the difference in K${}_{d}$ and stoichiometry values of two fits for labeled BCA I are discussed in Section 2.2.3.

Thermodynamic parameters that have been obtained for the respective binding mechanisms at 25 °C are reported in Table 2. Figure 5 shows an increase in the dissociation constants for both complexation reactions with temperature which supports the TDFRS measurements. Both ligands show a stoichiometry of 1:1 binding to the protein.

Increase in fluorine substitution is found to enhance the inhibitor power of sulfonamide ligands [60], implying that the more fluorine substituted ligand (PFBS) exhibits a higher association with BCA I, which is reflected by a lower K${}_{d}$ value, compared to 4FBS for all temperatures. Note that the dissociation constants of two ligands differ for BCA I only by a factor of 2.5, while for BCA II a factor of 25 has been reported [40,41].

#### 2.2.3. Measurements with a Thermophoretic Microfluidic Cell

We also used a thermophoretic microfluidic cell for measuring Soret coefficients [27]. This requires the system to be fluorescent labeled to determine the concentration profile. Details of the chemical structure of the dye (cf. Figure S2 in the Supporting Information) used for labeling and the procedure are given in the Supporting Information (cf. Section S3). Since the dye binds at a slightly higher pH = 8.3, we performed additional TDFRS measurements at this pH with the labeled and unlabeled protein. We found $S_{T}=0.028\pm 0.001$ K${}^{-1}$ and $S_{T}=0.032\pm 0.001$ K${}^{-1}$ for the unlabeled and labeled protein, respectively. Note, that the Soret coefficients measured at pH = 8.3 are roughly an order of magnitude smaller than at pH = 7.4 ($S_{T}=0.216\pm 0.003$ K${}^{-1}$). The reason might be that with increasing pH, the solute becomes more negatively charged and can form more hydrogen bonds, which often leads to lower $S_{T}$-values [12]. The Soret coefficient $S_{T}=0.018\;$K${}^{-1}$ measured in the microfluidic cell is roughly 40% lower than the TDFRS-value and has a high uncertainty. The measured fluorescence intensity is at the detection limit due to the low fraction of labeled proteins and decays due to photo bleaching. From repeated successful measurements we determine an uncertainty of 0.003 K${}^{-1}$, but the real error might be higher due to systematic errors caused by bleaching.

**Figure 6:**
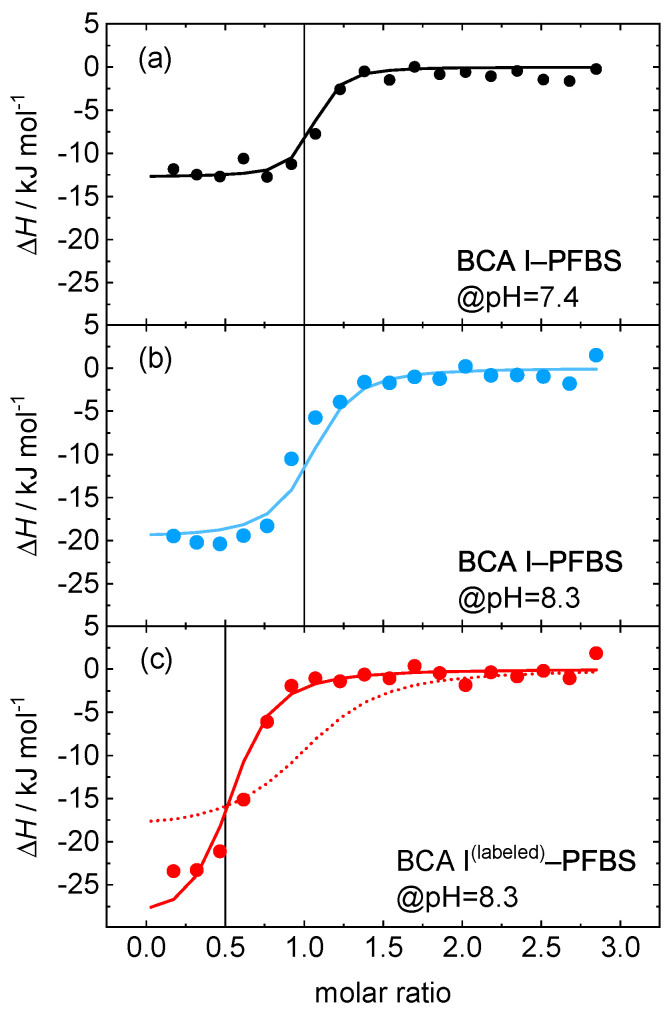
Molar change in enthalpy versus mole ratio of ligand over protein. (**a**) BCA I– PFBS at pH = 7.4 (**b**) BCA I–PFBS at pH = 8.3 and (**c**) the fluorescently labeled BCA I–PFBS at pH = 8.3. Dotted and solid lines corresponds to a fit with the stoichiometry of binding m=1 and m=0.5, respectively. All measurements have been performed at 25 °C.

To check the influence of the fluorescent label on the binding constant, we performed also ITC measurements. Since a change in pH is reported to affect the inhibitory power and activity of sulfonamides and protein, changes in the binding parameters are expected (cf. Figure 6) [61,62]. An increase in pH, shows a decrease in association of PFBS with BCA I (cf. Figure 5). Baronas et al. [63] report a weak increase in the dissociation constant of carbonic anhydrase with primary sulfonamides when the pH changes from 7.4 to 8.3. Once the protein is labeled, the association is only 30% compared to that of the unlabeled free protein at pH 8.3, so that we assume the dye blocks the binding site of the ligand (cf. Figure 5). Additionally, the stoichiometry of ligand:protein changes from 1:1 to 1:2. A hypothesis for this behavior could be the existence of protein dimer, thus a single ligand binding to two proteins as it has been previously reported for lysozyme [64]. Further experiments, e.g., using fluorescent correlation spectroscopy would have to be conducted to support this hypothesis. It has to be noted here that the $K_{d}$ value reported for labeled BCA I–PFBS binding has an higher uncertainty due to the low protein concentration obtained after fluorescent labeling. More details are given in the Supporting Information (cf. Figure S8 in Section S7).

In conclusion, we refrained from more systematic measurements of fluorescently labeled proteins due to the change of the binding process and the high uncertainty in the microfluidic cell.

### 2.3. Validation of the Relation between Soret Coefficient and Gibb’s Free Energy

This section mainly focuses on validating Equation (4) at two different temperatures which connects ΔG obtained from ITC with $S_{T}$ obtained from TDFRS. In the forthcoming sections, the calculation corresponds to T${}_{\mathrm{high}}=30$°C and $T_{\mathrm{low}}=20$°C.

#### 2.3.1. EDTA–CaCl${}_{2}$ System

As mentioned before, the first system that we chose for the validation of the derived mathematical expression is EDTA–CaCl${}_{2}$. With the $S_{T}$ values of EDTA, CaCl${}_{2}$ and the complex measured at T${}_{\mathrm{high}}$ and T${}_{\mathrm{low}}$, we have access to the change in Gibb’s energy (ΔΔG) of the individual components. On the basis of our observations, we calculate ΔG (30 °C) to be −36.5 ± 1.2 kJ mol${}^{-1}$, whereas from ITC measurements we obtained −36.4 ± 0.8 kJ mol${}^{-1}$. Both values agree within the error limits. Repeating the calculations for other temperature pairs lead also to an agreement within 10% (cf. Table S1 in Supporting Information).

#### 2.3.2. Protein–Ligand System

Now we apply the same procedure to the protein-ligand systems. In Table 3 we compare the calculated ΔG and the measured $\Delta G_{\mathrm{ITC}}$. For both the ligands values agree well within the error bars. Values for other temperature pairs can be found in the Supporting Information (Tables S2 and S3 in Supporting Information).

## 3. Materials and Methods

### 3.1. Sample Preparation

#### 3.1.1. EDTA–CaCl${}_{2}$ System

Stock solutions of EDTA and CaCl${}_{2}$ were prepared in 2-(N-morpholino)ethanesulfonic acid (MES) buffer of 10 mM, pH 5.8. EDTA solution of 1 mM and CaCl${}_{2}$ of 10 mM were used for measurements. For TDFRS samples, these solutions were filtered (0.2 μm) to remove dust particles. The transparent solution was filled into an optical quartz cell (Hellma) with an optical path length of 0.2 mm. For ITC measurements, a calibration kit (Malvern Panalytical) was used as received.

#### 3.1.2. BCA-Ligand System

To prepare the ligand and protein solutions, a sodium phosphate buffer (NaP buffer, pH 7.4, 20 mM) was used. Concentration of BCA I and ligand solutions were determined using UV-Vis absorption spectroscopy. Calibration curves (absorbance vs concentration) for BCA I, PFBS, and 4FBS were prepared starting from the stock solution of 1 mg/mL and measuring the absorbance maxima at 280, 268, and 257 nm, respectively. For BCA I, the concentration of the solution was reconfirmed using molar extinction coefficient of BCA I (51.0 × 10${}^{3}$ M${}^{-1}$ cm${}^{-1}$) and absorbance measured at 280 nm [65]. For TDFRS experiments BCA I and ligand concentrations of 10 μM and 110 μM were used. For ITC experiments, the same concentration was used for BCA I–PFBS system, while for BCA I–4FBS we had to increase protein and ligand concentrations to 20 μM and 300 μM, respectively.

### 3.2. Methods

#### 3.2.1. Thermal Diffusion Forced Rayleigh Scattering

Thermodiffusion of all the systems was measured by infrared thermal diffusion forced Rayleigh scattering (IR-TDFRS) [66,67]. This method uses the interference grating of two infrared laser beams (λ=980 nm) to generate a temperature grating inside an aqueous sample due to the inherent absorbtion of water at 980 nm [68]. A third laser beam (λ=633 nm) is refracted by this grating and the intensity of the refracted beam is measured. The intensity is proportional to the refractive index contrast of the grating, showing a fast rise over time due to the thermal gradient, then a slower change of intensity due to diffusion of the solute along the temperature gradient. The Soret, thermal diffusion and diffusion coefficient can be determined from the measurement signal when the refractive index contrast factors ${\left(\partial n/\partial c\right)}_{p,T}$ and ${\left(\partial n/\partial T\right)}_{p,c}$ are known [66].

#### 3.2.2. Contrast Factor Measurement

The change of refractive index with concentration ${\left(\partial n/\partial c\right)}_{p,T}$ was measured by a refractometer (Abbemat MW Anton Paar) at a wavelength of 632.8 nm. Refractive indices for five concentrations at six different temperatures (20–45 °C with a 5 °C gap) were measured to determine ${\left(\partial n/\partial c\right)}_{p,T}$. The concentration dependence of *n* was linearly fitted to derive the slope ${\left(\partial n/\partial c\right)}_{p,T}$ for all measured temperatures. The refractive index increments with temperature ${\left(\partial n/\partial T\right)}_{p,c}$ were measured interferometrically [69]. Measurements were performed over a temperature range of 20–45 °C, with a heating rate of 1.6 mK/s.

#### 3.2.3. Isothermal Titration Calorimetry

This technique has been extensively used to measure the thermodynamic parameters associated with protein–ligand binding interactions [70]. When a ligand binds to a protein, thermodynamic potentials (ΔG, ΔH, ΔS) change which can be measured by highly sensitive calorimetry. All other conventional methods measures binding affinity where as ITC measures the enthalpic and entropic contributions to binding affinity. This technique uses step wise injection of one reagent into the calorimetric cell. The working principle of the instrument has been discussed in the literature [26,70,71].

The calorimetric experiments for our study were performed with a MicroCal PEAQ ITC (Malvern Panalytical). For experiments on the reference system, EDTA (0.1 mM) in MES buffer (pH 5.8, 10 mM) was titrated with CaCl${}_{2}$ (10 mM) in the same buffer at 6 different temperatures (20–45 °C with 5 °C gap). A typical experiment consisted of 19 injections, 2 μL each. The time interval between injections was 2.5 min. Measurements were conducted 2 times with a new stock solution of EDTA and CaCl${}_{2}$ received from Malvern Panalytical. The same protocol was followed for BCA I–ligand sytems with concentrations as mentioned in Section 3.1.2. For protein–ligand systems, measurements were also recorded at 6 temperatures between 20 and 45 °C at pH 7.4. Additionally, to study the effect of pH and labeling, extra measurements were carried out for BCA I–PFBS system. Binding of this system was monitored at 25 °C for two scenarios: (a) BCA I–PFBS at pH 8.3 and (b) labeled BCA I–PFBS at pH 8.3. Data were analyzed using a single-site binding model subtracting background enthalpies, whereas ΔH and $K_{d}$ are treated as adjustable parameters.

#### 3.2.4. Thermophoretic Microfluidic Cells

The thermophoretic microfluidic cell can be either operated with large colloids (>500 nm), which are visible under the microscope or with fluorescently labeled macromolecules. The cell was made of PMMA and consisted of three channels [27]. We created a 1D temperature gradient in the measurement channel between the heating and cooling channels. In order to measure the temperature and concentration profile in the channel, a confocal microscope (Olympus IX-71 with a FV3-294 confocal unit) is used. A pulsed laser (λ=485 nm) was used for probing the fluorescence intensity and lifetime. The fluorescence intensity for the concentration of proteins was measured by a photomultiplier and the fluorescence lifetime in the measurement channel was characterized by fluorescence lifetime imaging microscopy (FLIM) using a correlator and a photomultiplier. The sample concentration of protein (BCA I) in the solution was 20 μM. The labeled protein content was 2.2 μM, which corresponds to 11% of proteins in the solution.

## 4. Conclusions

The main goal of this work is to investigate whether it is possible to connect thermodynamic parameters obtained by ITC with thermodiffusion parameters determined by IR-TDFRS. For a low molecular weight reference system, EDTA–CaCl${}_{2}$ and the protein BCA I with two ligands 4FBS and PFBS we were able to relate Soret coefficients with the Gibb’s free energies measured at two different temperatures with ITC using an empirical expression suggested by Eastman [29]. For all temperature pairs that have been studied for the aforementioned systems, the Gibb’s free energy values of the protein systems calculated agree within 8% with the values measured by ITC, which corresponds to 2-times the uncertainty of the ITC measurements. In the case of the system EDTA–CaCl${}_{2}$ the agreement is with 3% well within the uncertainty of the ITC measurement. This implies that Soret coefficients measured at different temperatures can be used to predict the Gibb’s free energy at other temperatures. This newly developed connection can be utilized to open promising gates in the accurate acquisition of the underlying binding and molecular dissociation mechanisms from the studied systems, if it is combined with molecular dynamic simulations [72] or complementary data obtained by AFM [73].

A second goal was to compare the results of the thermophoretic behavior of the protein and the complex with those obtained in a recently developed thermophoretic microfluidic cell. Fluorescent labeling of the protein is required to monitor the protein concentration using the thermophoretic microfluidic cell. For the here investigated protein BCA I, the fluorescent labeling influences the binding interactions severely so that we refrained from systematic thermophoretic measurements of the complex in the thermophoretic microfluidic cell. This is performed more efficiently with an intrinsic fluorescent protein, e.g., green fluorescent protein (GFP) or lysozyme. To gain a deeper microscopic understanding of the process, it would be desirable to perform neutron scattering experiments to determine the entropic contributions of the protein, thus unraveling the entire process [74,75].

Further, we found, that the Soret coefficients of EDTA and the EDTA–CaCl${}_{2}$ complex show an unusual temperature dependence that cannot be described by Equation (1). Of particular note is the abrupt drop in the Soret coefficient of EDTA between 25 and 30 °C. One finds some studies in the literature that also indicate a change in the behavior of EDTA in the same temperature range, but the database is insufficient to draw clear conclusions. At this point more systematic pH-dependent measurements also of other chelating agents, such as diethylene triamine penta-acetic acid (DTPA) or hydroxyethylethylenediaminetriacetic acid (HEDTA) would be desirable.

## Figures and Tables

**Table 1:** Thermodynamic parameters of the binding reaction between EDTA and CaCl${}_{2}$ measured using ITC at different temperatures by setting the stoichiometry of binding m=1 for the fit.

Temperature (°C)	K${}_{d}$ (nM)	ΔH (kJ/mol)
20	510.0 ± 49.0	−17.0 ± 0.3
25	623.0 ± 70.3	−17.2 ± 0.8
30	699.0 ± 55.5	−17.3 ± 0.7
35	852.0 ± 78.9	−17.6 ± 0.5
40	1210.0 ± 123.0	−17.8 ± 0.5
45	1570.0 ± 134.0	−18.0 ± 0.8

**Table 2:** Thermodynamic parameters of the binding reactions measured using ITC at 25 °C.

System	K${}_{d}$ (nM)	ΔH (kJ/mol)	ΔG (kJ/mol)
BCA I–PFBS	127.0 ± 47.2	− 12.5 ± 0.8	−37.4 ± 2.8
BCA I–4FBS	325.0 ± 58.7	−32.7 ± 0.4	−37.5 ± 1.3

**Table 3:** Comparison of ΔG that has been calculated and that has been measured with ITC.

System	T${}_{\mathrm{high}}$ (°C)	T${}_{\mathrm{low}}$ (°C)	ΔG${}_{\mathrm{calculated}}$ (kJ/mol)	ΔG${}_{\mathrm{ITC}}$ (kJ/mol)
BCA I–PFBS	30	20	−40.5 ± 1.1	−40.4 ± 1.3
BCA I–4FBS	30	20	−39.9 ± 3.9	−38.2 ± 1.5

## Data Availability

The data presented in this study are available on request from the corresponding author.

## Supplementary Materials

The following supporting information can be downloaded at: https://www.mdpi.com/article/10.3390/ijms232214198/s1.

## Abbreviations

The following abbreviations are used in this manuscript:

4FBS	4-fluorobenzenesulfonamide
BCA	Bovine Carbonic Anhydrase
EDTA	ethylenediaminetetraacetic acide
ITC	isothermal titration calorimetry
MES	2-(N-morpholino)ethanesulfonic acid
PFBS	Pentafluorobenzenesulfonamide
TDFRS	Thermal diffusion forced Rayleigh scattering
