# Supplementary material for: Complementary Experimental Methods to Obtain Thermodynamic Parameters of Protein Ligand Systems
Source: Int J Mol Sci. 2022 Nov 17;23(22):14198. doi: 10.3390/ijms232214198 (PMC9692857; doi:10.3390/ijms232214198)
Supplement: Supplementary file 1 [file ijms-23-14198-s001.zip › ijms-2041034-supplementary.pdf]

SUPPORTING INFORMATION: Complementary experimental methods to obtain thermodynamic parameters of protein ligand systems

Shilpa Mohanakumar, Namkyu Lee
and Simone Wiegand

Contents

S1 Mathematical relation between Soret coefficient and Gibb's free energy **S2**

S2 Various existing forms of EDTA in MES buffer **S4**

S3 Protein-ATTO 532 dye labeling **S4**
S3.1 Labeling BCA S4
S3.2 BCA purification S5

S4 Temperature dependence of the thermal diffusion D_T and diffusion coefficient D for protein-ligand systems **S6**

S5 Refractive index increments with temperature **S8**

S6 Data analysis and fitting of ITC measurements **S10**

S7 Analysis curves of ITC measurement for labeled BCA I with PFBS **S12**

S8 Validation of the relation between Soret coefficient and Gibb's free energy at other temperatures **S13**
S8.1 EDTA–$CaCl_2$ S13
S8.2 Protein-ligand S13
S8.2.1 BCA I–PFBS S13
S8.2.2 BCA I–4FBS S13

S1 Mathematical relation between Soret coefficient and Gibb's free energy

Soret coefficient and Gibb's free energy at different temperatures have been measured by TDFRS and ITC, respectively. A relation between S_T starts from a relation originally proposed by Eastman [1] and later rewritten by Würger [2] in a modern nomenclature,

$$S_\mathrm{T} = \frac{1}{k_\mathrm{B}T}\frac{dG}{dT} \tag{S1}$$

The Soret coeficients $S_\mathrm{T}^\mathrm{low}$ and $S_\mathrm{T}^\mathrm{high}$ correspond to T_low and T_high, respectively. Assuming a linear T-dependence of S_T with T, we write

$$S_\mathrm{T}(T) = (S_\mathrm{T}^\mathrm{low} + \Delta S_\mathrm{T})T \tag{S2}$$

with

$$\Delta S_\mathrm{T} = S_\mathrm{T}^\mathrm{high} - S_\mathrm{T}^\mathrm{low} \tag{S3}$$

Integration of Eq. S1 with respect to temperature in the range from T_low to T_high leads to

$$\int_{T_\mathrm{low}}^{T_\mathrm{high}} dG = \int_{T_\mathrm{low}}^{T_\mathrm{high}} k_\mathrm{B}TS_\mathrm{T}(T)dT \tag{S4}$$

using Eq. S2 we obtain

$$\Delta\Delta G = k_\mathrm{B}(S_\mathrm{T}^\mathrm{low} + \Delta S_\mathrm{T})(\frac{T_\mathrm{high}^2 - T_\mathrm{low}^2}{2}) \tag{S5}$$

For a mole of molecules holds

$$\Delta\Delta G = N_\mathrm{A}k_\mathrm{B}(S_\mathrm{T}^\mathrm{low} + \Delta S_\mathrm{T})(\frac{T_\mathrm{high}^2 - T_\mathrm{low}^2}{2}). \tag{S6}$$

S_T values of ligand, macromolecule and the complex at two different temperatures, T_high and T_low can be measured with TDFRS. $\Delta\Delta G$ corresponding to ligand, macromolecule and the complex can then be calculated with Eq. (S6). For systems like EDTA–$CaCl_2$ and protein-ligand for which binding is not strong, there are chances to find free ligands and macromolecules in the solution. In such cases, S_T(complex) will have also contributions from the individual compounds that are remaining in the solution. Hence there will be two main contributions to the S_T measured;

1. Contribution from the complex

2. Contribution from free macromolecule or free ligand

S_T(complex) for such systems has to be calculated excluding the contribution from free protein/free ligand (whichever is present in the system). It has to be noted that in EDTA–$CaCl_2$ system, EDTA acts as the macromolecule (in the cell of ITC) and $CaCl_2$ as the ligand (injected through a syringe into the cell of ITC). Dissociation constant K_d measured by ITC is;

$$K_{\mathrm{d}} = \frac{[\text{protein}][\text{ligand}]}{[\text{complex}]} \tag{S7}$$

where [protein], [ligand] and [complex] are the concentrations of free protein, free ligand and complex, respectively. Since the total concentration of protein (protein(total)) and ligand (ligand(total)) is known, Eq. (S7) can be rearranged as follows,

$$K_{\mathrm{d}} = \frac{[\text{protein(total)} - [\text{complex}]][\text{ligand(total)} - [\text{complex}]]}{[\text{complex}]} \tag{S8}$$

Solving the quadratic equation Eq. (S8) gives access to [complex], from which we can calculate the fraction of the free protein f_{P} and ligand f_{L} accordingly,

$$f_{\mathrm{P}} = \frac{[\text{protein(total)} - [\text{complex}]]}{\text{protein(total)}} \tag{S9}$$

$$f_{\mathrm{L}} = \frac{[\text{ligand(total)} - [\text{complex}]]}{\text{ligand(total)}} \tag{S10}$$

$$f_{\mathrm{C}} = 1 - (f_{\mathrm{P}} + f_{\mathrm{L}}) \tag{S11}$$

Analysis of K_d values indicates that for EDTA–$CaCl_2$ and both protein-ligand systems, the fraction of free ligand (or $CaCl_2$) is higher than that of the free protein (or EDTA) (at 25°C, for BCA I–4FBS system, fraction of free protein and ligand are 0.008 and 0.7, respectively). Hence for the further calculations, we neglect the contribution from the free protein. We propose the following equation for the S_T(total) that is measured using our IR-TDFRS setup :

$$\frac{1}{S_{\mathrm{T}}^{\text{total}}} = \frac{1 - f_{\mathrm{C}}}{S_{\mathrm{T}}^{\text{complex}}} + \frac{f_{\mathrm{L}}}{S_{\mathrm{T}}^{\text{ligand}}} \tag{S12}$$

where $S_{\mathrm{T}}^{\text{total}}$ is the S_T measured using IR-TDFRS, $S_{\mathrm{T}}^{\text{ligand}}$ is the S_T of ligand, f_{C} is the fraction of complex and f_{L} is the fraction of free ligand. Rearranging Eq. (S12) leads to

$$S_{\mathrm{T}}^{\text{complex}} = \frac{1 - f_{\mathrm{C}}}{\frac{1}{S_{\mathrm{T}}^{\text{total}}} - \frac{f_{\mathrm{L}}}{S_{\mathrm{T}}^{\text{ligand}}}} \tag{S13}$$

Access of S_T(complex) at T_{high} and T_{low} enables the calculation of $\Delta\Delta G$ corresponding to the complex. Obtained values can be used to establish a relation between ITC and TDFRS measurements as shown in the main manuscript.

S2 Various existing forms of EDTA in MES buffer

EDTA ($C_{10}H_{16}N_2O_8$) exists in several forms in MES buffer [3]. EDTA is a hexadentate ligand which has six lone pairs of electrons that are available for binding. Protonation enables EDTA to exist in various states ranging from a fully protonated (Fig.S1a) to fully deprotonated stage (Fig.S1b). It can also exist in some intermediate stages between these two forms with one to three protons. Experimental studies confirm that $EDTA^{4-}$ and $HEDTA^{3-}$ exist, playing a crucial role in forming complexes with the metal ion [4, 5, 3]. It has to be noted that the complexation reaction primarily proceeds through $EDTA^{4-}$ forms, but $HEDTA^{3-}$ reacts also with a lower probablity [3]. The majority form depends on the pH (cf. Fig.1 [4, 5] and Fig.9 in [3])

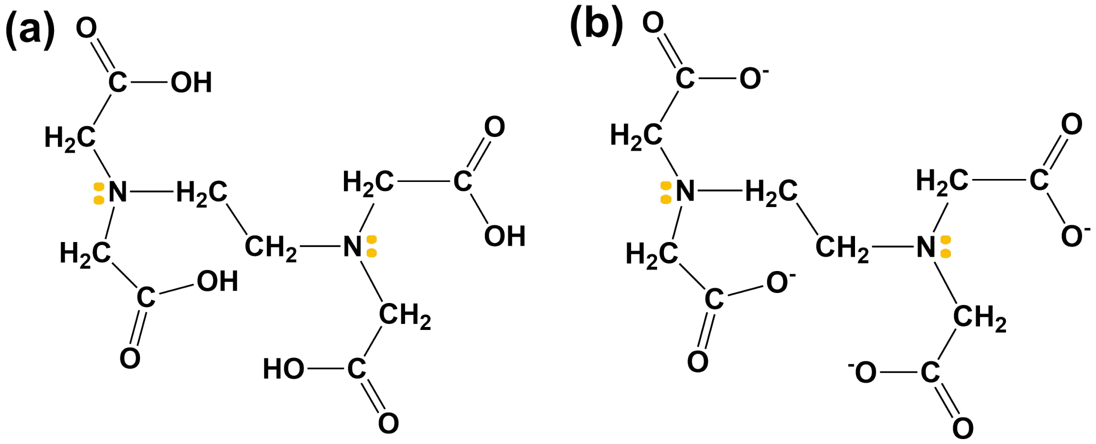

Figure S1: (a) Protonated form of EDTA (b) Deprotonated form of EDTA

S3 Protein-ATTO 532 dye labeling

S3.1 Labeling BCA

ATTO 532 dye (cf. Fig.S2) was used for labeling Bovine Carbonic Anhydrase (BCA) I. Protein standard (100 μM) was prepared in sodium phosphate

buffer (20 mM). For NHS-ester labeling the pH of the sample was adjusted to 8.3 by adding drops of a $NaHCO_3$ solution (2.1 g in 50 ml water). 1 mg of ATTO 532 was dissolved in 200 μl of dimethyl sulfoxide (DMSO) which is used as the standard solvent for the dye. The dye solution was mixed with the protein solution so that the final concentration ratio dye:protein is 200 μM:20 μM in 1 ml of the solution. The rest of the volume was filled up with buffer solution of pH 8.3. The sample were kept in shaker overnight for thorough mixing.

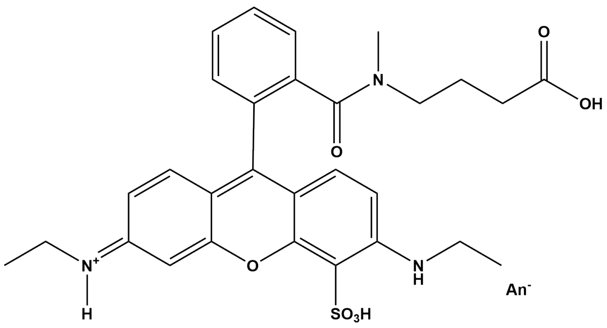

Figure S2: Chemical structure of ATTO 532 dye

S3.2 BCA purification

The following procedure was followed to remove the unbound dye. Zeba™ Spin-Säulen columns were used for the purification of the mixture. Once the column is settled after centrifugation (1000 rpm for 2 minutes), the process was repeated with buffer of pH 7.4. 300 μl of the mixture was transferred to the column and purified. Absorption of the solution was measured using UV-VIS spectrophotometer. Labeled proteins have an absorption at 532 nm, which gives the corresponding concentration.

S4 Temperature dependence of the thermal diffusion D_T and diffusion coefficient D for protein-ligand systems

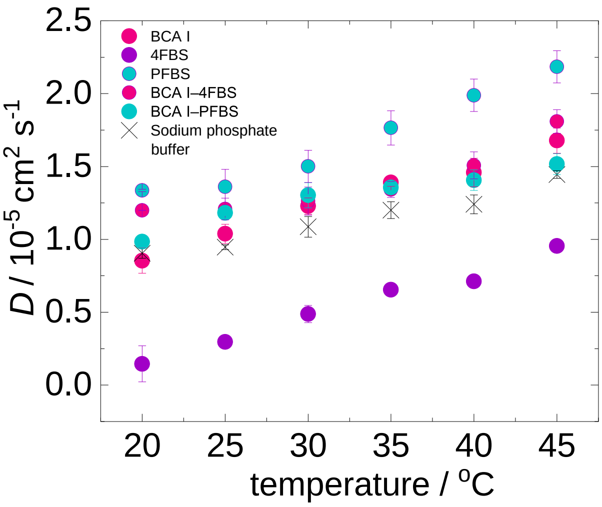

Figure S3: Temperature dependence of D for BCA I (protein), 4FBS and PFBS (ligands), corresponding protein-ligand complex, sodium phosphate buffer

Dependence of diffusion coefficients on temperature is shown in Fig.S3. Diffusion of protein gets faster when ligand binds to it. This could be related to the fact, that the complex is less hydrophilic, so that it can move faster compared to the free protein, which shows the slowest diffusion. The increase in D with temperature due to decrease in viscosity can also be seen in Fig.S3. Dependence of thermodiffusion coefficient on temperature is shown in Fig.S4. here it is striking that the thermal diffusion coefficients of the complexes and the ligands are very similar, which D_T is much lower, which could also be related to the higher hydrophilicity of the free protein.

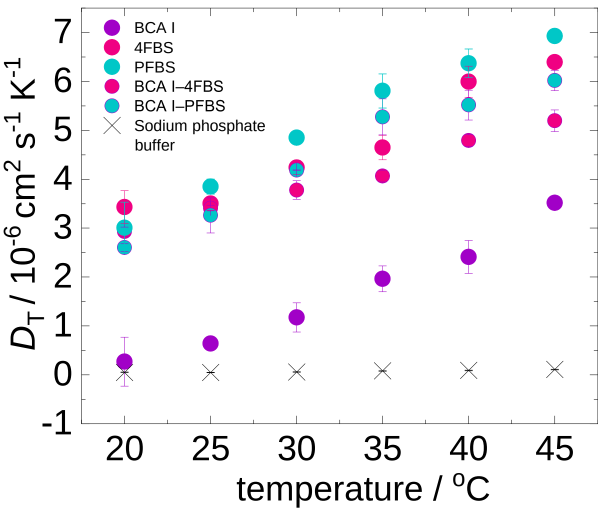

Figure S4: Temperature dependence of D_{T} for BCA I (protein), 4FBS and PFBS (ligands), corresponding protein-ligand complex, sodium phosphate buffer

S5 Refractive index increments with temperature

Figure S5 and S6 shows the refractive index increments for EDTA–$CaCl_2$ and both protein-ligand systems.

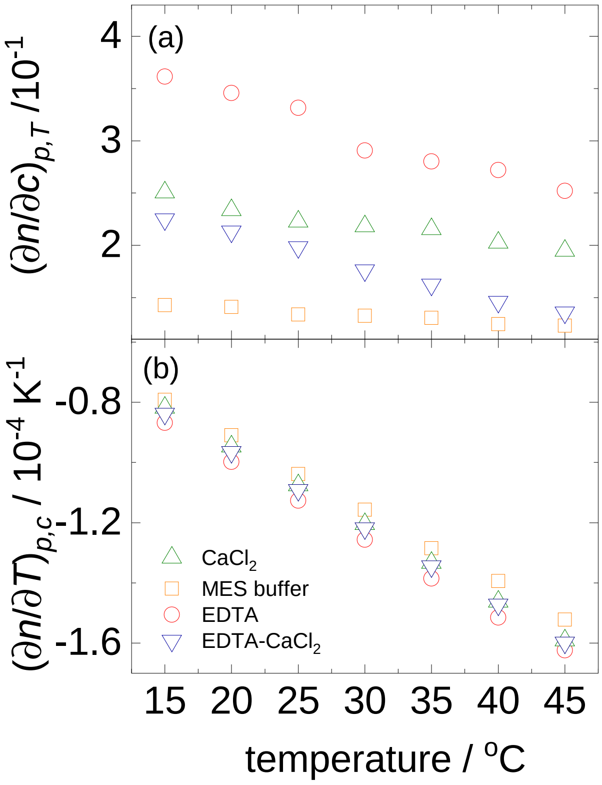

Figure S5: (a)Temperature dependence of $(\partial n/\partial c)_{p,T}$ for EDTA–$CaCl_2$ system (b)Temperature dependence of $(\partial n/\partial T)_{p,c}$ for EDTA–$CaCl_2$ system

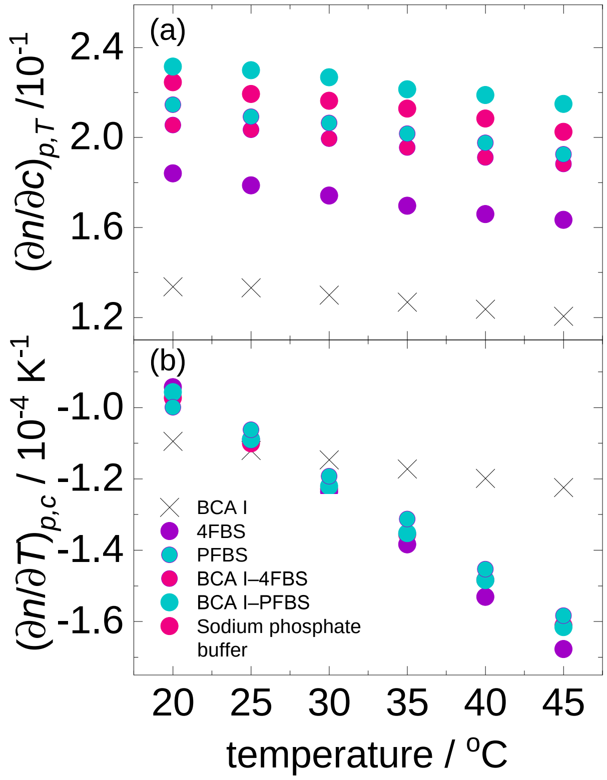

Figure S6: (a)Temperature dependence of $(\partial n/\partial c)_{p,T}$ for protein-ligand systems (b)Temperature dependence of $(\partial n/\partial T)_{p,c}$ for protein-ligand systems

S6 Data analysis and fitting of ITC measurements

We have used the most common binding model (1:1 binding) for the analysis of our ITC data. The following notations are used further: m is the number of binding sites, θ is the fraction of sites occupied by ligand X, M_t and M are the bulk and free concentration of macro molecule, X_t and X are the bulk and free concentration of ligand, ΔH is the molar heat of ligand binding, V_0 is the active cell volume. Binding constant,

$$K = \frac{\theta}{(1-\theta)X} \tag{S14}$$

Total ligand concentration,

$$X_t = X + m\theta M_t \tag{S15}$$

Combining Eq. S14 and Eq. S15;

$$\theta^2 - \theta[1 + \frac{X_t}{mM_t} + \frac{1}{mKM_t}] + \frac{X_t}{mM_t} = 0 \tag{S16}$$

The total heat content of the solution Q at a given time for an ITC measurement is

$$Q = m\theta M_t \Delta H V_0 \tag{S17}$$

Solving Eq. S16 for θ and substituting it in Eq. S17 gives,

$$Q = \frac{mM_t\Delta HV_0}{2}[1 + \frac{X_t}{mM_t} + \frac{1}{mKM_t} - \sqrt{(1 + \frac{X_t}{mM_t} + \frac{1}{mKM_t})^2 - \frac{4X_t}{mM_t}}] \tag{S18}$$

Once an injection of ligand into the cell occurs, software calculates the heat associated with the injection. What is of primary interest for the study is the change in heat content of ith injection to that of $i-1^{th}$ injection. Change in heat after i^{th} injection is given by;

$$\Delta Q(i) = Q(i) + \frac{dV_i}{V_0}[\frac{Q(i)+Q(i-1)}{2}] - Q(i-1) \tag{S19}$$

where dV_i is the correction factor introduced to compensate the heat contribution from the displaced volume. This is because, for each injection there is a volume of protein-ligand complex that is expelled from the cell which is identical to the volume of ligand that is injected. Fitting involves initial guesses of m, ΔH and K which allows to calculate $\Delta Q(i)$ for each injection. This is then compared with the experimental $\Delta Q(i)$ which is measured and then improving m, ΔH and K values until the best fit. A typical ITC measurement curve and molar enthalpy curve is shown in Fig.S7.

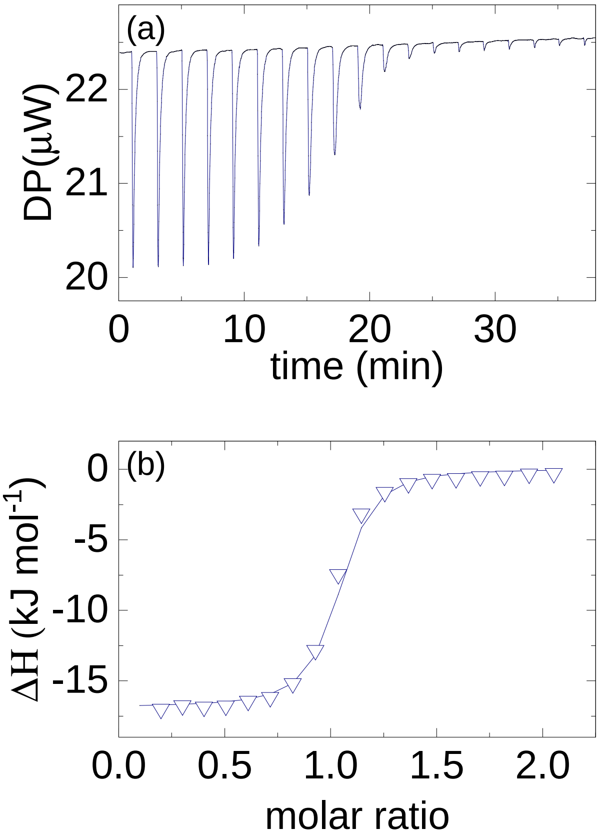

Figure S7: (a)Raw data output for EDTA–$CaCl_2$ binding reaction measured at 25°C (b)Integrated data output for EDTA–$CaCl_2$ binding reaction measured at 25°C plotted as molar change in enthalpy against molar ratio

S7 Analysis curves of ITC measurement for labeled BCA I with PFBS

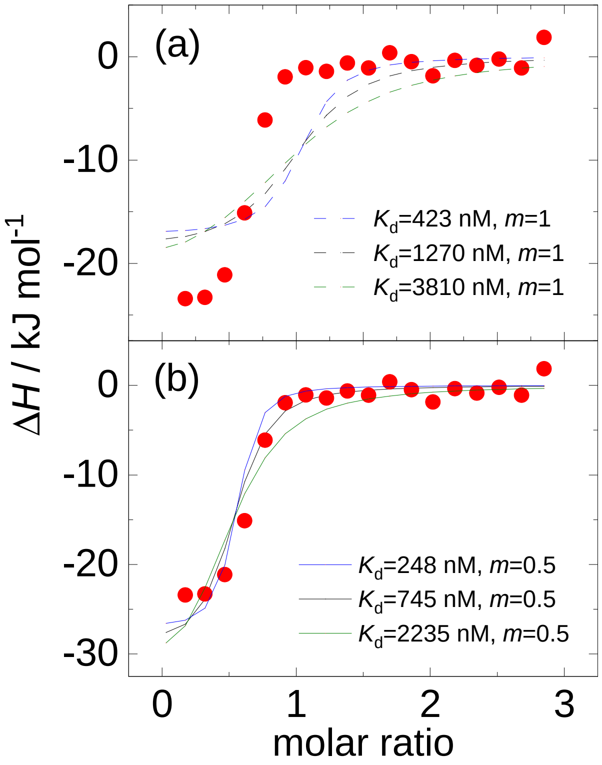

Figure S8: Integrated data output for labeled BCA I–PFBS binding measured at 25°C. (a) and (b) corresponds to the fit obtained with m=1 and m=0.5, respectively. The black line corresponds to the optimum fit generated by the analysis software with K_{d}(optimum). The blue line corresponds to the fit obtained by fixing K_{d} to a three times lower value of K_{d}(optimum) and green line corresponds to the fit obtained by fixing K_{d} to a three times higher value of the optimum fit K_{d}(optimum). Lower and higher K_{d} values used for the fits are listed in the legend.

Fixing the value of K_{d} and m adjusting ΔH gives the results shown in Fig. S8. Changing the value of K_{d} changes the shape of the curve, but the data are not compatible with $m = 1$. For $m = 0.5$ the lower K_{d}-value gives also a reasonable description, which implies that the uncertainty of K_{d} is at least 50%.

S8 Validation of the relation between Soret coefficient and Gibb's free energy at other temperatures

ΔG calculated at higher temperatures using Eq. 4 (cf. Sec.1 of main manuscript) and its comparison to the ITC measurements will be discussed for all systems in the following paragraphs.

S8.1 EDTA–$CaCl_2$

Table S1 lists the calculated $\Delta G_{calculated}$ and ΔG_{ITC} measured with ITC for EDTA–$CaCl_2$. Both values agree within the error bars.

Table S1: Table enlists ΔG values that are calculated and measured using ITC at different temperature combinations for EDTA–$CaCl_2$ system

T_{high} (°C)	T_{low} (°C)	$\Delta G_{calculated}$(kJ/mol)	ΔG_{ITC}(kJ/mol)
20	30	-36.5 ± 1.2	-36.4 ± 0.8
25	35	-36.1± 3.2	-35.5 ± 1.8
30	40	-35.7 ± 3.4	-34.8 ± 2.4
35	45	-33.3 ± 1.4	-34.2 ± 1.9

S8.2 Protein-ligand

S8.2.1 BCA I–PFBS

Table S2 lists the calculated $\Delta G_{calculated}$ and ΔG_{ITC} measured with ITC for BCA I–PFBS. Both values agree within the error bars.

S8.2.2 BCA I–4FBS

Table S3 lists the calculated $\Delta G_{calculated}$ and ΔG_{ITC} measured with ITC for BCA I–PFBS. Both values agree within the error bars.

Table S2: Table enlists ΔG values that are calculated and measured using ITC at different temperature combinations for BCA I–PFBS system

T_{high} (°C)	T_{low} (°C)	$\Delta G_{calculated}$(kJ/mol)	ΔG_{ITC}(kJ/mol)
20	30	-40.5 ± 1.1	-40.4 ± 1.3
25	35	-44.0 ± 2.6	-46.8 ± 0.6
30	40	-48.2 ± 3.1	-52.1 ± 1.2
35	45	-54.9 ± 2.9	-55.9 ± 1.1

Table S3: Table enlists ΔG values that are calculated and measured using ITC at different temperature combinations for BCA I–4FBS system

T_{high} (°C)	T_{low} (°C)	$\Delta G_{calculated}$(kJ/mol)	ΔG_{ITC}(kJ/mol)
20	30	-39.9 ± 3.9	-38.2 ± 1.5
25	35	-36.6± 3.4	-39.4 ± 1.1
30	40	-40.3 ± 1.8	-40.7 ± 0.8
35	45	-45.5 ± 3.5	-42.1 ± 1.3

References

[1] E. D. Eastman. Theory of the Soret effect. *J. Am. Chem. Soc.*, 50:283–291, 1928.

[2] A. Würger. Is Soret equilibrium a non-equilibrium effect? *C. R. - Mec.*, 341:438–448, 2013.

[3] J. Reiter. *CFD analysis of EDTA-$CaCl_2$ reaction in a microfluidic channel to aid in design of novel calorimeter device*. PhD thesis, Northeastern University Boston, Massachusetts, 2015.

[4] R. J. Kula, G. H. Reed. Nuclear magnetic resonance investigation of ligand exchange kinetics in the calcium(II)-EDTA system. *Analytical chemistry*, 38:697–701, 1966.

[5] J. D. Carr and D. G. Swartzfager. Kinetics of the ligand exchange and dissociation reactions of calcium-aminocarboxylate complexes. *J. Am. Chem. Soc.*, 97:315–321, 1973.
